# Series: Public engagement with research. Part 1: The fundamentals of public engagement with research

**DOI:** 10.1080/13814788.2023.2232111

**Published:** 2023-08-14

**Authors:** Steven Blackburn, Megan Clinch, Maarten de Wit, Albine Moser, Jette Primdahl, Esther van Vliet, Christine Walker, Fiona Stevenson

**Affiliations:** a Institute of Applied Health Research, University of Birmingham, Birmingham, UK; b Centre for Public Health & Policy, Queen Mary University of London, London, UK; c Patient Research Partner Stichting Tools, Amsterdam, the Netherlands; d Department of Family Medicine, CAPHRI School for Public Health and Primary Care, Maastricht University Medical Centre, Maastricht, the Netherlands; e Department of Regional Health Research, University of Southern Denmark, Denmark; f Academic Collaborative Centers, Knowledge Transfer Office, Tilburg University, Tilburg, the Netherlands; g Research User Group, Primary Care Centre Versus Arthritis, School of Medicine, Keele University, Keele, UK; h Primary Care and Population Health, University College London, London, UK

**Keywords:** Public engagement, patient and public involvement, primary care, research

## Abstract

**Background:**

In the first of a four-part series, we describe the fundamentals of public engagement in primary care research.

**Objectives:**

The article’s purpose is to encourage, inform and improve the researcher’s awareness about public engagement in research. For a growing number of researchers, funders and patient organisations in Europe, public engagement is a moral and ethical imperative for conducting high-quality research.

**Discussion:**

Starting with an explanation of the role of public engagement in research, we highlight its diversity and benefits to research, researchers and the public members involved. We summarise principles of good practice and provide valuable resources for researchers to use in their public engagement activities. Finally, we discuss some of the issues encountered when researchers collaborate with members of the public and provide practical steps to address them. Case studies of real-life situations are used to illustrate and aid understanding.

**Conclusion:**

We hope this article and the other papers in this series will encourage researchers to better consider the role and practice of public engagement and the potential added value to research that collaborating with the public could provide.

KEY MESSAGES

- Public engagement can support high-quality, relevant and impactful primary care research.
- We share principles of good practice of public engagement and provide useful resources to support engagement activities.
- Public engagement is not always easy. We provide practical steps to address challenges faced when collaborating with the public.

## Introduction

In many European countries, public engagement is seen by research funders and regulators as an essential aspect of research. It can support high-quality and relevant research to improve primary health services and benefit patients [1–3]. An important feature is the experiential insights of people with specific health conditions and symptoms. This helps ensure that the focus and outputs of research are relevant to the public [1,2,4].

Increasingly, there is more demand for primary healthcare which is evidenced based [5]. We need to do relevant research that will make a difference in peoples’ lives. As such, there are ethical and democratic reasons why engaging the public in the research process is warranted [6]. These include:

**A democratic right**: Public money funds most research, so the public has a right to have a say on what research is done and how it is conducted.
**An ethical right**: As health research involves human participants and/or their data, the public should have a say on how people take part safely in studies, plus how their data is accessed and used.
**Public accountability:** Ensuring that research is value for money and beneficial to society by including public members in research commissioning and governance.

There is much variability in how researchers conceptualise and embrace patient engagement in their research. While part of the research culture in some places, partly driven by a political and public interest agenda, many researchers are unfamiliar with the value of public engagement in research. Also, some may lack the knowledge and skills to engage with the public meaningfully and effectively. On the other hand, for a growing number of researchers, funders and patient organisations in Europe, public engagement is a moral imperative for conducting high-quality research. We hope this article (and others in this series) will encourage researchers to consider the role and potential added value to research that collaborating with the public provides.

As the first of a four-part series, this article provides an overview of the fundamentals of public engagement in research. Case studies of real-life situations are used to aid understanding. First, we explore what public engagement is and the benefits of primary care research. Then we discuss seven issues and challenges to consider when planning public engagement.

The authors include people with lived experience of primary health care services, researchers and public engagement practitioners, all with an interest in and experience of engaging with the public in research.

## What is public engagement with research?

### Definitions

Public engagement is when members of the public, communities and/or public organisations have an active role in shaping, conducting, and sharing research and its findings. At its core is a mutually respectful relationship and beneficial partnership between researchers and the public.

Several theoretical frameworks explain the different roles and engagement approaches (see Supplementary File 1) [7–9]. There is no global consensus on terminology or definition of public engagement. We use the UK-based National Coordinating Centre for Public Engagement’s definition of public engagement as it encapsulates the broad range of engagement approaches and contexts discussed in this series (Box 1) [10].

Box 1 Definition of public engagement used in this series

‘Public engagement describes the myriad ways in which the activity and benefits of higher education and research can be shared with the public. Engagement is by definition a two-way process, involving interaction and listening, intending to generate mutual benefit.’

National Coordinating Centre for Public Engagement [13]

Collaborating with the public might be a mindset change for some researchers and public members. In public engagement, public members are viewed not as research participants or a source of data but as active contributors to the research process. They represent a unique source of knowledge that adds value to the research process - providing expertise based on people’s lived experiences.

### Approaches to public engagement

**Figure 1.**
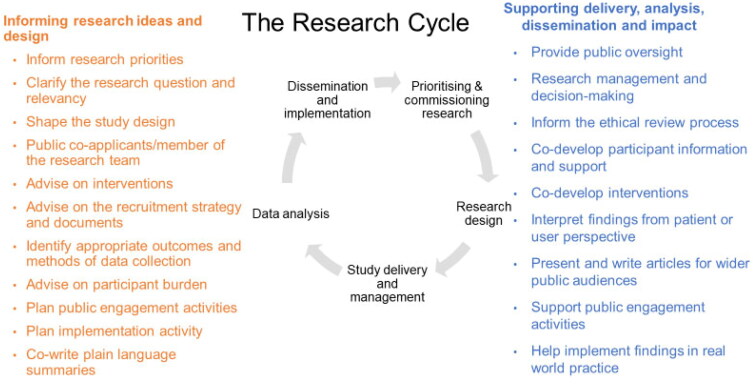
The different roles of public contributors throughout the research lifecycle.

Researchers can work with the public in many aspects of a research study throughout its lifecycle (Figure 1). There is no formula for how or when researchers can engage with the public. Public contributors can have active roles in all types of research (from basic lab and translational research studies to clinical and real-world trials), during any part of a study or throughout the whole research process. This includes sharing research with non-academic audiences through contributing to dissemination plans and materials for the public and supporting the use of research in practice [11]. However, we acknowledge that there can be aspects of some research studies (e.g. quantitative data analysis) where the potential role and value of public engagement are less straightforward. Nevertheless, we feel researchers should always consider engaging with the public when feasible, using all the skills and advice available to make it successful.

Some approaches to public engagement are based on consultation methods, e.g. discussion groups, and public meetings. Other approaches are more collaborative, enabling greater two-way dialogue between the public and researchers. Here, public members have an active role in the research process. For example, as part of patient advisory groups giving insights on specific elements of a study, or a research team member involved in making decisions about the design and delivery of a study [12].

Research can be co-produced by researchers and the public or even be led by public members with the support of researchers [13]. In these studies, public members share ownership and responsibility in the design and delivery of research. The third part in this series explores the coproduction of research and the role of people and communities who lead research studies.

Public engagement with research also includes sharing and discussing the findings and implementing the knowledge gained from research with the public. Approaches might include citizen’s panels and juries, science festivals and broadcast/social media. Public members are often involved in discussing the implications of research findings for different communities and shaping how best to communicate them with the wider general public.

Researchers can embrace different and creative ways to engage with the public. No single approach is likely to meet the needs of all research studies, nor the needs of the public members involved. Nevertheless, many researchers actively involve the public in their research at the earliest opportunity (e.g. setting the priorities for research, choosing the research topic/question) in open and inclusive ways and continue to engage closely with the public throughout the research. Valuable opportunities for gaining relevant public insight to influence the conception and design of studies are lost when researchers come with an almost final research plan and ask public members or organisations for general feedback or to provide a letter of endorsement.

## What are the benefits of public engagement?

### Benefits to research studies

Some benefits of public engagement in research studies might not be anticipated. These might be seemingly insignificant suggestions to how a participant information sheet is worded or a major change in a recruitment process based on the insights of someone with lived experience. A research design that ‘looks good on paper’ may not be practical for a given context. Not all public contributions, however, will result in a change in a research process or decision – but having confirmation (or a ‘reality check’) that a particular research process is acceptable to patients is also reassuring and worthwhile.

In summary, the benefits include:

*Helping to decide the important and relevant research questions to ask* by combining experiential input from patients with the clinical and academic knowledge of clinicians and researchers. Doing so will more likely address the issues that matter most to the public and healthcare providers [14].
*Making the research more inclusive, accessible and acceptable to patients, carers and service users* by providing practical ideas of who, how and where to recruit research participants. Co-developing clear and uncomplicated participant information materials with public contributors will enhance the accessibility of research and helps participants better understand what they need to do – thus supporting better recruitment and retention [3]. Working with public members to co-design more user-friendly interventions and ensuring that the study outcomes measured are important to patients will likely make the research more acceptable to participants.
*Ensuring that the delivery of research remains focussed on the needs of patients and the public* by involving public members in regular advisory committees throughout a project.
*Helping to interpret findings from a public perspective* to reflect issues that are important to patients, thus helping to provide more useful outputs for healthcare providers and the public [14].
*Raising public awareness, use and trust of research* through public contributors helps to share knowledge generated by research in accessible formats and languages so it can be discussed and used by patient groups and healthcare professionals (Explored further in Part 4).

### Benefits to researchers

Public engagement enables researchers to improve their understanding and empathy of people’s healthcare needs. It helps to have a genuine interest in engaging with the public and take time to work collaboratively with them. Engaging in open discussions with public contributors can offer a different way of thinking about a particular research issue or problem. It can help researchers develop new skills and confidence in group facilitation, communication, and collaborative working with different people. With support and recognition from mentors and supervisors, public engagement can help individuals become more rounded and better researchers [15].

### Benefits to public contributors

Contributors get the satisfaction that they are offering something back to society. It can give a sense of belonging to something important and giving to the greater good [16]. Making a positive difference in research that may ultimately lead to improvement in patients’ lives is a fundamental reason why many public members get involved with research in the first place.

People can develop new skills and confidence, along with a better understanding of their own health condition [17]. For some, public engagement has helped them live better with and self-manage their health. For others (known to the authors), it has enabled opportunities to return to work or experience new places.

## How is public engagement done well?

Several publications and toolkits guide research on how best to engage with the public [18–23]. In summary, good public engagement:

- Starts early and continued throughout the research.
- Is carefully planned and organised.
- Has a clear purpose.
- Is inclusive and open to a diversity of people.
- Allows time for developing relationships with public contributors and groups.
- Considers individual public contributors’ needs and provides the appropriate support, guidance and training.
- Communicates with public contributors in clear and plain language.
- Respects and acknowledge the contributions of public members to research.
- Reports on the contribution of and learning from public engagement (using the GRIPP2 reporting checklist) [24].

A personal account of good practice is provided in Case Study A (Supplementary File 2) by CW, a public contributor and co-author of this article.

### Evaluating public engagement

To support learning and improvement, researchers are encouraged to evaluate their public engagement. Many frameworks to support evaluation are available [25]. The need to assess the impact of public engagement on research is widely debated [26]. Current evidence is considered weak, which may lead to uncertainty about the value of public engagement. However, the view of public engagement by some as simply an *intervention* that can be *measured,* somewhat negates the complexity of the interaction between researchers and the public and the formative value of continuous reflection and learning [26].

## What are the challenges of public engagement?

Engaging the public with research can present several challenges and often does not occur at all [27]. Issues can arise from a lack of training and awareness [28], inadequate planning, inflexible institutional systems and processes [28], and/or a lack of early agreement between researchers and public members regarding how they will work together [27]. We discuss some of these issues and provide practical suggestions to address them; case studies provide real-life examples. They are based on the experiences of the co-authors, the public engagement community, and published examples.

### Challenge 1: Defining the role of public engagement

The role of public engagement depends on the type of research. In studies that rely on the recruitment of people into the study, use surveys, collect body samples or measurements from human participants, or test interventions with patients, public members can offer valuable experiential insight and advice on how research participants are recruited, communicated with and interact with researchers during a study [29].

In other studies, such as early-phase clinical studies or health data research, when there is no or very little direct interaction with people as research participants, researchers may find it difficult to realise the value of public engagement. Also, public engagement may not be required or relevant in every study phase. Patient charities and researchers have developed guidelines on the role and value of public engagement in lab-based research, including case examples (Box 2) [30].

Box 2 Value of public engagement in lab-based research

- Help to understand the value and importance of the research to wider society from a patient or user’s perspective
- Better focus on research areas that may ultimately benefit patients
- Consider potential issues of public contention or sensitivity in research that may be viewed as controversial
- Raise public awareness and discourse about the future potential outcomes of the research (e.g. a new treatment, cure or health knowledge)

On the other end of the participatory spectrum, community stakeholders work in ongoing partnerships with researchers to plan and design participatory research studies and interventions [31]. An exploration of participatory-based research and community-led research is provided in Part 3 of this series.

Regardless of the research phase, design and topic, it is important to identify the purpose of any public engagement activity, where the public can add value to the research and consider the preferences, life experiences and interests of the public members involved. Without careful consideration of these things at the outset, a public engagement activity risks becoming tokenistic. Moreover, some public contributors may be more comfortable in certain roles or activities than others. For example, someone who is very creative can help develop engaging and accessible communication material to share the findings of a study with the general public.

Whatever the role(s) of public contributors in a study, people must be enabled to provide a meaningful contribution to the research. This will require an investment in time, energy, and resources to support their involvement [18] (Box 3).

### Challenge 2: Identifying diverse public contributors with relevant lived experience

Finding members of the public to be involved in a primary care research study is not always straightforward. Identifying people with relevant lived experience may be difficult as there may not be organised patient groups for the many health conditions managed in primary care. Case Study B (Supplementary File 3) provides an example of this. Where patient groups do exist, they may not reflect the sociodemographic characteristics required [32]. Identifying public contributors and developing effective relationships takes time and effort [33]. This should not be underestimated during the planning of a research study. Case Study C (Supplementary File 4) illustrates the importance and benefits of this. We urge researchers to embrace a broad range of people with relevant lived experiences from different communities and societal sectors that reflects the intended study population. This will help to ensure that the research is informed by a diversity of relevant public perspectives. Engaging with a smaller group of people is sometimes more realistic and certainly better than no engagement at all. The involvement of at least two public contributors is recommended [18]; however, involving more people will enable a diversity of opinions and contributions. There are several routes to invite public contributors, such as from people who have taken part in a study previously, existing patient organisations or community groups, or *via* advertising (e.g. posters, social media). Note, however, that many patient organisations across Europe get inundated with requests from researchers and they often lack the capacity to provide support. People could be invited from clinic lists too. Though, caution ought to be taken to avoid potential conflicts of interest. For example, patients might feel obliged to be involved if they are approached directly by their treating physician/clinician.

Some researchers have the support of an institution’s public engagement team, who may already have links with patient and community groups. Other researchers may need to ‘get out there’ themselves and start making new relationships. Research organisations should also address any systematic inequalities and barriers, including cultural competency, to promote equitable opportunities to engage with groups, especially those from under-served communities [22]. The importance of creating a more inclusive environment for engaging with people who experience inequalities is discussed in detail in Part 2 of this series (Box 4).

### Challenge 3. Enabling equitable dialogue and contribution between public contributors and researchers

Meaningful public engagement is a two-way interaction between public members and researchers. However, researchers often involve people within a largely transactional process where researchers set the agenda for how the public can be involved. This is not entirely surprising given the strict timelines and protocols dictating research development and delivery. Nevertheless, providing time and spaces for more equal interactions and dialogue will allow better sharing of ideas, perspectives and knowledge. This takes patience and flexibility from all partners [35]. This issue is accentuated in co-produced studies, which require the sharing of power and responsibilities with the public. Co-production is explored further in Part 3 (Box 5).

### Challenge 4: Ensuring respectful communication and interactions

Most public engagement takes place with respect and civility. But as with any human interaction, there are (rare) occasions when things go wrong: communication breaks down, someone says something inappropriate or people refuse to cooperate. This can be highly uncomfortable for everyone involved and difficult to know how to react (Box 6). Case Study D (Supplementary File 5) provides real-life examples and lessons.

### Challenge 5: Engaging with people with physical, mental or learning impairments

Some groups of people may inadvertently be excluded if their accessibility needs are not considered. Group meetings with researchers (either face-to-face or virtual) may not be appropriate and comfortable for everyone. Researchers should consider carefully how they can make reasonable adjustments to enable and support people with physical, mental or learning impairments to contribute. Discussing how people would like to be involved early will certainly help (Box 7).

### Challenge 6. Recognising and rewarding the contribution of public members

Public contributors give their time and effort to collaborate with researchers. Many researchers recognise public contributions to studies in publications [24]. In some countries, offering financial compensation (or some other reward) to public contributors is recommended [23]. At the very least, their expenses should be reimbursed (e.g. travel, accommodation, broadband). However, navigating institutional finance departments and understanding the impact of payment on some individuals’ benefit claims can be problematic [35] (Box 8).

### Challenge 7: Do I need ethical approval for public engagement?

In many countries, there is no requirement to seek research ethics approval for public engagement, nor ask public contributors to provide formal informed consent [36]. However, there may be instances when public engagement raises certain ethical considerations and/or confidentiality issues [37]. For example, when research is conducted on the public engagement activities itself, if engagement is with vulnerable people or settings, or if public members have direct access to research participants and/or data. On these occasions, researchers should seek advice from an ethics committee on whether approvals may be needed (Box 9).

## Conclusion

In summary, public engagement is regarded as a moral imperative that supports high-quality research. It can provide many benefits to research and the people involved. Effective and meaningful public engagement is best achieved with careful planning and discussion with the public contributors to agree on how they will be involved. Engaging with the public should not be an afterthought or tokenistic – or solely a means to secure research funding. It is done to make the research more relevant and appropriate to the people the research is focused on. The added value provided by public engagement is not always obvious, it’s not entirely measurable - but even a subtle change to a research protocol suggested by a public contributor can make an important difference to the success of a study. The process of public engagement can be challenging but can be met with the practical steps outlined in this article. Doing public engagement well will likely improve your research, broaden your research skills and ensure that health research is more equitable and beneficial to all.

Box 3 Practical steps to define the role of public contributors

- Discuss and agree on how and why the public will be engaged in different aspects of a research study.
- Agree on a set of ground rules (or ‘ways of working together’) at outset of the engagement activities.
- Provide simple and clear role descriptions for the public members, including how and when they will be involved and how the research team will communicate with them

Box 4 Practical steps to identify people with relevant lived experiences

- Identify people who have relevant lived experience that reflect the intended study population
- Reach and go out to groups and communities
- Have early and informal discussions with a small group of people you intend to engage with to address how best to approach engagement activities.
- Advertise opportunities for involvement widely, using different formats and media
- Get the support of intermediaries who already have links with the individuals or groups.
- Invest time and resources to develop sustainable relationships with individuals and groups
- Carefully plan and get advice on suitable meeting times, duration, format and location to make engagement activities as inclusive as possible.

Box 5 Practical steps to enable equitable dialogue

- Create safe and welcoming environments.
- Dress informally and avoid titles of authority to introduce yourself (e.g., “Dr” and “Professor”)
- Jointly discuss and decide with public contributors on the most desirable and feasible format of engagement.
- Engage in active two-way conversations about a research topic or process, and avoid a formal ‘interview or focus group’ format
- Provide people with appropriate support and guidance to help them understand basic elements of research and give them the confidence to contribute effectively.
- Having a dedicated person(s) to offer support and help to public contributors before, during and/or after their involvement
- Make sure the public contributors include all those who should be involved are included as early as possible
- Ensuring that all public contributors and researchers understand that their contributions are of equal importance will enable joint ownership of key decisions

Box 6 Practical steps to ensure respectful communication

- Agree on ground rules for working together, including how disputes will be resolved
- Offer guidance for all on respectful communication
- Outline people’s expectations at the outset
- Address inappropriate behaviour early to prevent others in the group from getting offended and losing trust in the team
- Talk separately with the individual(s) to better understand the cause of possible inappropriate behaviour, recap the ground rules and agree to an appropriate next step (including discontinuing someone’s involvement if an agreeable solution cannot be found)

Box 7 Practical steps to enable and support people with physical, mental or learning impairments

- Avoid this being an issue by considering the needs of all individuals involved
- Explore different ways of engaging with people, how and where people want to engage with you and what will work for them.
- Be patient, creative and flexible in how you engage with people (e.g., use visual, text-based, audio and arts-based approaches)
- Get support from colleagues with relevant experience and/or professional expertise in working with specific groups of public members

Box 8 Practical steps to recognise and reward the contribution of public members to research

- Recognise and acknowledge the contribution of public members in research outputs (e.g., use the GRIPP2 checklist [36])
- Develop a clear, simple policy and straightforward process for paying public contributors. Engage with your institutional finance departments early to ensure feasibility
- Agree to a suitable level and method of remuneration with the public contributors
- Include expenses of public contributors in the budget of the research funding proposal (e.g., compensation, transportation, costs of caring responsibilities, etc.)
- Ensure that public contributors’ expenses are reimbursed promptly

Box 9 Practical steps to consider possible ethical aspects of public engagement

- Think whether your public engagement raises any possible ethical concerns
- Seek advice from an ethics committee if in doubt
- Consider confidentiality or data protection issues. If so, public contributors may need to sign a confidentiality and/or data sharing agreement

## Supplementary Material

Supplementary File 5

Supplementary File 4

Supplementary File 3

Supplementary File 2

Supplementary File 1
