# Supplementary material for: Series: Public engagement with research. Part 1: The fundamentals of public engagement with research
Source: Eur J Gen Pract. 2023 Aug 14;29(1):2232111. doi: 10.1080/13814788.2023.2232111 (PMC10431741; doi:10.1080/13814788.2023.2232111)
Supplement: Supplementary File 5 [file IGEN_A_2232111_SM0906.docx]

## **Supplementary File 5**

### Case Study D: Ensuring clear communication between researchers and public contributors / patient organisations

**What happened**

When working with a research charity it became apparent that the expectations that the public contributors had in terms of involvement in data collection and leading on writing research publications did seem to match those of the research team.

**Consequences**

Resistance to involvement of trained researchers in data collection meant the data collected was not of the highest quality. When a publication was drafted for an academic paper outlining the key results and experts by experience were asked to comment and contribute but not lead, the experts by experience were upset.

**Lessons learnt**

When setting up a project, be clear about the expectations of all involved and if working with a research charity as an intermediary, take particular care to ensure clear communication with all involved.
