# Supplementary material for: Series: Public engagement with research. Part 1: The fundamentals of public engagement with research
Source: Eur J Gen Pract. 2023 Aug 14;29(1):2232111. doi: 10.1080/13814788.2023.2232111 (PMC10431741; doi:10.1080/13814788.2023.2232111)
Supplement: Supplementary File 4 [file IGEN_A_2232111_SM0903.docx]

## **Supplementary File 4:**

###

### Case Study C – Recruitment and selection of a patient contributor

**What happened?**

When initiating collaboration with two patient research partners, a research nurse introduced them to their role. The introduction encompassed clarification of the role of the patient research partners and the kind of knowledge and experience the researchers expect from them. At the first meeting with the research team, the tasks the patient research partners wanted to become involved in, were discussed and defined. This included how to communicate (mail, telephone, face-to-face meetings) and how often or how much time they were able to dedicate. The tasks could be development of the final protocol, funding applications, development of patient information and consent material, how and when to approach eligible participants for the study, discussion of findings, participation as co-author and the later presentation of findings and implementation. Regular evaluations took place with the research nurse and the principal investigator during the study.

**Consequences**

The patient research partners felt recognised; they contributed positively at meetings and by e-mail on various written materials. They wanted to participate in developing patient information, discussing findings as co-authors and in the later implementation. Evaluations have revealed that the patient research partners often thought they did not contribute with anything special – ‘just their experiences,’ whereas the researchers could tell them that this was exactly the very valuable contributions they needed, and this dialogue was critical to both parties.

**Lessons learnt**

Introduction, evaluation and feedback during a study on the importance of the contributions from the patient research partners are important elements. A culture where it is expected to involve patient research partners in all studies and where a dedicated person can help introduce and evaluate the process is constructive.
