# Supplementary material for: Series: Public engagement with research. Part 1: The fundamentals of public engagement with research
Source: Eur J Gen Pract. 2023 Aug 14;29(1):2232111. doi: 10.1080/13814788.2023.2232111 (PMC10431741; doi:10.1080/13814788.2023.2232111)
Supplement: Supplementary File 3 [file IGEN_A_2232111_SM0901.docx]

## **Supplementary File 3**

### Case Study B – Identifying public contributors with relevant lived experience

**What happened?**

An orthopaedic PhD student was told that she was required to involve patients in her study on total-knee replacements in osteoarthritis. Without knowing where to look for eligible patients, she approached a rheumatologist on the team who suggested a young and enthusiastic person with rheumatoid arthritis with whom he had positive experiences.

**Consequences**

The young researcher invited the patient representative to every team meeting. After six months, the patient felt disappointed. She told the researcher *‘I have nothing with this project, and I feel very lonely. I am 32 years. I have no problems with my knees, and I can’t identify myself with older people with osteoarthritis. I am of no use in this study.’* The patient stopped her involvement.

**Lessons learnt**

This case study demonstrates that researchers need guidance on organising meaningful engagement of patients in scientific research. Senior researchers are responsible for facilitating patient engagement by preparing their staff and students to do this properly. The case also shows how important it is to ensure that patient representatives do have lived experience with the condition or subject under study. Finally, patients often indicate that they prefer to work with one or more fellow patients. Having a sparring partner makes it easier to prepare for meetings, it ensures a more diverse perspective and makes involvement in research more enjoyable.
