# Supplementary material for: Series: Public engagement with research. Part 1: The fundamentals of public engagement with research
Source: Eur J Gen Pract. 2023 Aug 14;29(1):2232111. doi: 10.1080/13814788.2023.2232111 (PMC10431741; doi:10.1080/13814788.2023.2232111)
Supplement: Supplementary File 2 [file IGEN_A_2232111_SM0893.docx]

## Supplementary File 2

### Case Study A: A personal account of good public engagement practices by a public contributor (CW, co-author)

In the early stages of developing a research idea the principal investigator will bring together a group of patient contributors. The contributors listen to the proposal and give helpful, experiential views, opinions and ideas to add benefit to the proposal’s aims. They are encouraged to be constructive so the proposal can encompass a broad range of areas in its bid for funding.

This group often meets up again as the proposal moves through its developmental progress.

As the proposal begins to take shape and before submission of complete documentation, patient co-applicants are approached and join the research team, contributing throughout its journey to eventual funded status and implementation. They assist with the production of patient-facing documentation and in the writing of the plain language summary at the beginning of the formal proposal. They join the team meetings and contribute their ideas and views from the patient’s perspective. They stay with the team as the proposal progresses and assist with implementing and disseminating the eventual outcomes.

As the proposal reaches certain stages of its journey, it often becomes necessary to seek further views from the initial group of contributors on matters that require their opinions on the suitability of patient-facing documentation.

Payment for meeting attendances by public contributors will have been agreed by the principal investigator at the commencement of the proposal.
