# Supplementary material for: Series: Public engagement with research. Part 1: The fundamentals of public engagement with research
Source: Eur J Gen Pract. 2023 Aug 14;29(1):2232111. doi: 10.1080/13814788.2023.2232111 (PMC10431741; doi:10.1080/13814788.2023.2232111)

# Supplementary File

## Models of Engagement

##### Figure S1 Welcome Trust Public Engagement ‘Onion’[46]


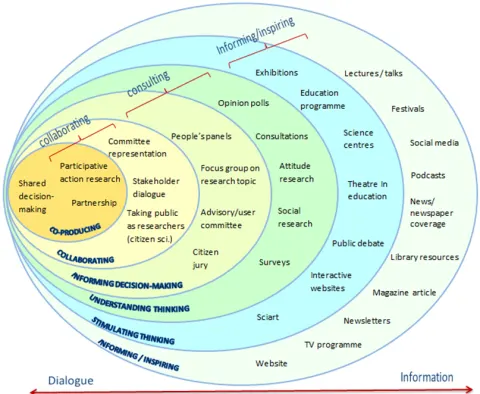


Figure S2 The Involvement Matrix[12]


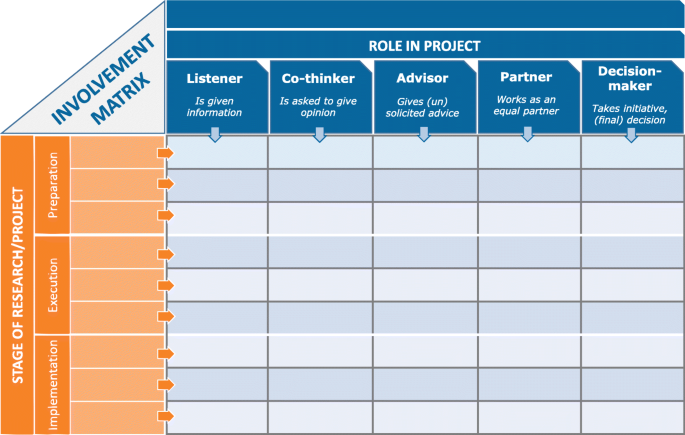


Figure S3 Cube theoretical framework for public involvement in research[11]


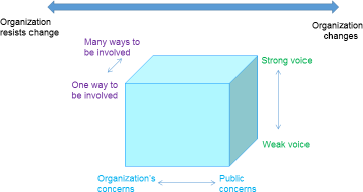

Supplement: Supplementary File 1 [file IGEN_A_2232111_SM0891.docx]
